# Socially responsible genetic research with descendants of the First Australians

**DOI:** 10.1186/2041-2223-3-22

**Published:** 2012-11-14

**Authors:** Sheila M van Holst Pellekaan

**Affiliations:** 1 School of Biotechnology and Biomolecular Sciences D26, University of New South Wales, Kensington, New South Wales, 2052, Australia

**Keywords:** Aboriginal, Australia, Colonization, Dispossession, Genetic, Ethics, Resistance

## Abstract

Aboriginal Australians, one of the world’s indigenous peoples now outnumbered through colonization, are the most under-represented in genetic research because they feel that the benefits do not outweigh the social cost of involvement. Descendants of the First Australians have survived a period of European occupation during which time they were dispossessed of land, language and cultural identity resulting in inequities in health, education, and employment opportunities. Compared to Maori and Native American peoples, the ability to form organizations that help to control their affairs is very recent. The desire to control is understandably strong yet the ‘gate-keeping’ role of some organizations risks shifting the control away from smaller communities and has become increasingly politicized. In the past, research practices by Western scientists were poorly presented and have resulted in resistance to proposals that are perceived to have no beneficial outcomes for participants. In this age of advanced technological expertise in genetics, benefits to all humanity are clear to those carrying out research projects, yet not always to those being asked to participate, presenting extra challenges. Excellent guidelines for ethical conduct in research are available to assist researchers, prospective participants, and ethics committees or review boards that approve and monitor procedures. The essence of these guidelines are that research should be carried out with a spirit of integrity, respect, reciprocity, parity, recognition of survival and protection of social and cultural values, a need for control and shared responsibility. Specific Aboriginal organizations, with which researchers need to work to negotiate partnerships, vary within and between Australian states and will always expect Aboriginal personnel to be involved. People experienced in the consultation process are necessary as part of a team. By working patiently through lengthy negotiations with Aboriginal Australians, scientists can achieve valuable results, but failure to do so with respect and understanding will not yield hoped for outcomes. My own experience working with communities in the Darling River region of western New South Wales has been an enriching and rewarding one, with a long period of successful research lately delayed by increased expectation of monitoring and involvement at state level.

## 

Aboriginal and Torres Strait Islander peoples of Australia are very sparsely represented in published genetic studies. Yet it is well recognized through these and from archaeological, anthropological, and linguistic evidence that they are one of the world’s longest living cultural groups, having occupied the continent for at least 40,000 to 50,000 years. The situation in which descendants of the First Australians find themselves today as a minority in their own land is not unique, yet of all the world’s indigenous peoples, resistance to genetic research is possibly strongest. This arises from many things, possibly a combination of various circumstances within broader contexts: a more recent colonial history (than, for example, Native Americans who had 200 years of the colonial experience before Europeans settled in Australia in 1788) within an age of accelerated technological advancement, and even more recently, proper recognition of their place in Australia where societies at large experience increased politicization of human rights and social issues. There are examples worldwide where researchers are able to work effectively with indigenous peoples, taking time to understand the process that is required to achieve collaboration. As one of these researchers I appeal to colleagues in the scientific community to appreciate the challenges and the pitfalls involved. I also ask indigenous peoples, especially those in positions of influence on committees and review boards, to appreciate the structures and constraints under which scientists have to operate and to avoid increasing the bureaucratic complexity of monitoring research to a level that effectively results in exclusion from potentially beneficial involvement. This is currently the case in many parts of Australia.

The social impact of Western science on all peoples has been profound. Technological advances have generated enormous power to affect people lives with clear advantages, yet also many costs to cultural and social wellbeing. Over the last decades, genetic technology has reached an unprecedented level of power to probe the origin and evolution of living organisms. For indigenous peoples of the world, who survive in environments that were changed forever by colonizing ‘others’, extra challenges arise. Increasingly, genetic researchers seek to describe and compare complete human genomes to understand diversity in living populations and from human remains of the past. Only by including indigenous peoples in these studies can the genetic history of our species be properly understood. Application of knowledge derived from these data is necessary to answer questions of ancestral, evolutionary, demographic, medical, and forensic relevance yet most Aboriginal Australians find it difficult to appreciate the value that such research might bring.

Poor research practices of the past resulted in distrust that remains entrenched [1-3] even though salient lessons have been learnt from those mistakes by practicing researchers. Some responsibility for the lasting distrust of motives behind genetic studies arose from the presentation of the Human Genome Diversity Project (HGDP) in the 1990s to some Aboriginal Australian organizations. It is so well remembered today that the experience and publicity that it generated is frequently raised and has harmed the efforts of many Australian scientists who have worked hard with Aboriginal organizations and individuals to present the benefits of genetic research, to involve participants in projects and to deal together with the negative concerns [2]. Entrenched distrust is hard to overcome.

There are clear arguments in favor of describing genetic history of our species and scientists working in genetic research have, for the most part, proceeded with their work responsibly and with ethical integrity. Institutions and funding bodies have greatly improved their attention to the social impact of research, yet very few individual scientists fully appreciate the situation of indigenous peoples in the world of today, do not understand why resistance in Australia is so strong and are poorly equipped to deal with expectations, meet the challenges, and develop partnerships.

The long history of human habitation in the island continent of Australia, known from archaeology, linguistic, and anthropological studies, has been confirmed from several genetic studies most recently reviewed [4] and including several from my own research [5-8]. They have created strong interest within the scientific community. Over the last decades, many scientists outside Australia have sought access to samples as the range of diversity within Australia is not well-known and many language groups/regions are not represented. Even for the participants of some published studies, traditional links are unknown. While requests for samples may have clear scientific validity, appreciation of the situation for scientists working in Australia is poorly understood by those making the request and do not seem to understand that samples cannot be sent outside the country without specific consent under current ethical guidelines and acceptable protocols.

This article discusses the pertinent example of the challenges facing both genetic researchers and descendants of the First Australians in achieving partnerships that may realize the value of genetic research for all, without creating social disharmony.

## Historical background

The colonization experience is a familiar one for surviving indigenous peoples throughout the world. Lives for most are markedly affected by dispossession of culture, language, and traditional lifestyles after being forced to move aside to make room for large-scale expansion dominated by technological, political, and economic power. In New Zealand, the treaty of Waitangi signed in 1840 exerted a powerful influence on subsequent colonial events though it did not stop the continuing process of dispossession. Native peoples of the Americas suffered shameful exploitation by European colonizers and in many cases, the lands subsequently ceded to them bore minimal resemblance to their traditional connections. In Africa, Europe, and Asia, human demographic history has been marked by patterns of dispersal motivated by different needs ranging from changing circumstances such as climate, economic needs, population pressure, conflict, and the lust for power. Genetic researchers try to understand the genetic history of people today to answer questions related to biological and social wellbeing, yet frequently fail to appreciate the concerns of indigenous peoples as a result of colonial history, different world views, and a struggle to retain identity. An understanding of history is not only essential to facilitate interpretations of diversity, population sizes in the past, and geographic distribution of results from genetic studies on living people, but is fundamental to principles of ethical conduct.

In Australia, the colonization period is recent (from 1788) and indigenous marginalization began immediately on the east coast extending rapidly into the interior with exploration and pastoralization [6]. Unlike Aotearoa (New Zealand), there has never been a treaty and in terms of the law, land rights that are central to the identity of Aboriginal Australians, have been achieved only since the 1970s [9,10]. Social history for the descendants of the First Australians has been marked by dispossession, appropriation of land, separation of families, and exploitation of women. There is resultant resistance to research *per se*, in particular to genetic research [1-3,11] and an increased effort to gain control through indigenous organizations at the local, national, and international level where Australian Aboriginal representatives sit on United Nations advisory committees. It is well-known that many research practices (such as the collection of skeletal remains for museum collections) in the early part of the 20th century had little regard for the impact on Aboriginal Australians. After many years of negotiation, the engagement of the Australian Government in current efforts to return those remains to traditional kin is beginning to heal some of the hurt [12]. There is a place for genetic research in this process, but the legacy of the past has meant that, with some exceptions [13,14], few Aboriginal communities have agreed to the genetic study of ‘ancient’ human tissue.

It is well documented that by the time European settlers arrived in 1788, the Australian continent was entirely occupied by diverse and distinctive language groups. Under a doctrine of *terra nullius*, that occupation was not legally recognized until 1992 [15]. From the early 1800s, the forced relocation of whole families and the subsequent removal of children under ‘assimilation’ policies, sometimes to locations hundreds of kilometers from traditional country [16] meant that many living people with Aboriginal ancestry are still unsure of their traditional connections. In the changed circumstances of today, communities live in scattered populations and there is considerable movement between cities, rural, and remote regions [17]. While many Aboriginal and Torres Strait Islander people have overcome the social disadvantage of the past, it is not so for many rural and remote communities where opportunities for training and employment are sparse. With the emergence of Land Rights [9], Aboriginal Australians have made some gains in managing their own affairs, establishing organizations that have some influence on policies albeit not as much control that they feel is their right. For researchers this manifests as a wish for indigenous control over research projects that include Aboriginal participants and in some cases, hard fought funding has been lost due to delays or outright refusal to sanction non-indigenous involvement. I summarize some of my own experiences and the processes currently in place in Australia that scientists need to follow to work with Aboriginal Australian participation. Many of the principles behind these processes are those of fundamental human rights, yet need to be seen in the context of colonization that has lead to social disadvantage for many peoples who have been colonized.

## Practice in the Australian context

My own experience began in 1992 when I sought Aboriginal participation for a mitochondrial DNA (mtDNA) study and successfully obtained samples from people of the Paakintji (Barkindje), Ngyembaa, and Mutthi-Mutthi peoples of the Darling River region in western New South Wales. My own background in nursing, medical research, anthropology, and archaeology provided knowledge and experience for consultation and negotiation proceeding through the Local Land Councils and participant families [3]. Consent was initially for mtDNA analysis and later (1995 to 1997) consent was obtained to do health-related genetic studies. The mtDNA analysis [5-7] identified strong and Australian specific mt haplogroups now known as M42a, S, O, P4b, P8 which together with other studies have made a major contribution to knowledge of the matrilines in the region [4] already known to be a significant Pleistocene habitat. Of the genetic studies with Australian Aboriginal participation that have been published, my research is rare in being able to describe language group affiliation with accuracy, yet this information is fundamental to interpretation of genetic results about the arrival and dispersal of people in the Near Oceanic region. Nuclear work was carried out on various single candidate loci and one genome analysis [8], however progress has been very slow lately. During the last 20 years, many community visits have been made by me and some colleagues to the region. I have always visited organizations, individuals, and families, providing reports, maintaining good relationships, and obtaining letters of support from various organizations. However progress has been delayed lately due to increased requirements of ethics committees external to the local communities.

Institutional ethics approval was given throughout by the University of Sydney Human Ethics Committee, then University of New South Wales Human Research Ethics Committee (UNSWHREC), and state government health services. Since 2005, negotiations for ongoing research have proceeded through the Aboriginal Health and Medical Research Council Ethics Committee (AH&MRCEC) of New South Wales which was not in place at the beginning of my research, but arose from efforts to establish Aboriginal Community Controlled Health Services (ACCHS) during the 1990s. It is currently a requisite of the UNSWHREC to gain ethical approval from the AH&MRCEC [http://www.ahmrc.org.au/Ethics] before seeking institutional ethics approval for ongoing work. That has been delayed due to the requirements of the AH&MRCEC that involve revision and re-approval of current consent forms, renewed consent even for those that did so in 2011, the establishment (by the researcher) of another ‘reference group’ comprising Aboriginal people with expertise in health and genetics, organizational approval from the regional Aboriginal community controlled health organization who have stated unwillingness to be involved but expressed the right of individuals to participate. In addition, delays are due to the lengthy documentation of 20 years of research which leads to apparent misreading of information previously supplied. Additional difficulties for myself have been in the form of incorrect assertions (easily refuted) that have questioned my professional integrity, presumably arising from uninformed sources, external to the communities or families with whom I maintain very good relationships. In a separate multidisciplinary (funded) project in northern Australia, the population genetics that was planned with my involvement as one component was vetoed by a regional Land Council, despite community consent. These delays are extraordinarily difficult, yet I strongly believe that only by persevering will Aboriginal and Torres Strait Islander people realize that poor practices of the past do not continue in Australia and truly engage in what can be beneficial work.

These experiences indicate that geneticists who wish to work with indigenous peoples need to be prepared to build relationships that recognize social issues around the physical and cultural survival of communities. This must begin by contact, either direct or indirectly through an experienced collaborator, with an Elder group or local leaders in the relevant community. It is neither appropriate nor fair to ask national Aboriginal leaders to act as a go-between. That contact must be made in a manner that indicates respect for the history and values of that community. Guidelines for appropriate research with indigenous peoples in Canada [18], New Zealand [19], as well as with Aboriginal Australians have been in place for some time [20-24] and are constantly being updated. Most relevant resources for geneticists, because of the sensitivity to the use of DNA, are those that include guidelines for Aboriginal and Torres Strait Islanders who may wonder what they need to consider if asked to participate in research [25]. The intrinsic values common to the guidelines and explained more fully in the document, are that projects should be presented and negotiated with a spirit of integrity; respect; reciprocity; parity; recognition of survival and protection of social and cultural values; a need for control and shared responsibility. The essence of these guidelines for ethical research practice has been, for the most part, incorporated into submission guidelines for institutional ethics committees in Australia. Ethical approval for specific projects needs to be sought from one or more appropriate ethics committees in institutions such as universities, hospitals, research centers, or specific Aboriginal organizations, depending on the state or region. Forensic scientists collect samples for forensic purposes and reports that arise from these collections should have a forensic focus. The use of the samples and the data that arise are restricted to forensic science in accordance with regulations that are not the same in all Australian states.

The application of ethical principles in Australia will vary as communities may be from remote areas, rural towns with mixed populations or cities. However, the expectations will include appropriate *engagement*, *consultation*, and *negotiation* which will hopefully lead to participation. A useful resource for researchers, because it is also prepared for Aboriginal and Torres Strait Islander communities and individuals, details eight steps with tasks for researchers [25] under the following headings: building relationships; conceptualization or thinking and planning; developments and approval; data collection and management; analysis or looking at the meaning; report writing; dissemination; learning from the experience. The point about this general ‘plan’, which may seem obvious to scientists, is that researchers and participants have specific tasks and responsibilities at each step. For the researchers and for funding bodies, there is a clear need to invest resources into every step of the process and preparedness for personal engagement is essential. With regard to DNA research, there are special issues related to consent, custodianship of samples, personal information and data dissemination. These issues have formed the focus of discussions between geneticists, medical and social scientists, Aboriginal people, and ethics committee representatives over recent years and for the last 2 years at ‘Roundtable’ discussion groups hosted by the Lowitja Institute for which a freely downloadable document is available [23,24]. These documents are informative for researchers.

Human research ethics committees (or review boards) normally work toward approval for a specific project and named personnel that have been submitted to the committee according to the particular institution. Any variation to the project or personnel requires additional consent from the relevant committee. In genetic research, this means that samples that remain from one study cannot be passed to other scientists or used for further studies with different aims, without renewed consent. It is counterproductive to the efforts of the many people who are involved directly or indirectly in negotiated research with Aboriginal or Torres Strait Islander people in Australia, if researchers outside Australia proceed to use samples that were collected in another era. It is expected that renewed consent to use those samples should be obtained.

The requirements of human research ethics committees/review boards are to provide evidence from the research team (usually through the principal investigator) that adequate care has been taken to follow the principals outlined in available guidelines (albeit respecting the confidentiality between researcher and participant). This will include declarations that appropriate information has been supplied and that individuals have given consent on forms approved by the particular committee, say for example the University of New South Wales Human Research Ethics Committee (UNSWHREC). Where an additional ethics committee is involved such as the Aboriginal Health and Medical Research Council Ethics Committee (AH&MRCEC) of New South Wales, there is an expectation that evidence of community consent to a project is in the form of a letter or document from the appropriate organization [22]. This frequently raises the issue of whether community engagement means that communities/organizations want to give consent. In the region in which I work, the answer is no.

## Unresolved issues

Several of these have caused and are still causing great delays in my own work and, in my view, result in the foundering of beneficial partnerships between other geneticists and Aboriginal and Torres Strait Islander peoples.

• Control of research by Aboriginal and Torres Strait Islander people is understandable against the historical background that generated disadvantage yet it is interpreted to varying degrees. In an increasing number of communities, substantial direct involvement is in place especially for projects that have a direct impact on day to day life. However, for some Aboriginal organizations or ‘peak bodies’, control is expected to be more than involvement in a project. For example, the NSW AH&MRCEC advises that there should be

‘Aboriginal community control over all aspects of the proposed research, including research design, ownership of data, data interpretation, and publication of results’ [22].

For some types of projects such as genetic research that require the use of expensive institution-centered equipment, technology and data analysis expertise, a collaborative approach is necessary as none of us have the full range of expertise required. Very few Aboriginal and Torres Strait Islander people express interest in careers in science and to achieve the degree of control envisaged, the available qualified Aboriginal and Torres Strait Islander people have to take on extra responsibilities. To attract more people into research, greater incentives in the form of scholarships/education and career opportunities need to be offered. My own research has been criticized by the AH&MRC because it has only ‘involved’ communities and families by personal visits with constant feedback, but has not had the resources to employ a community member as a part of the project. This issue of control has prevented some well-designed projects from eventuating.

• Community consent for a project is appropriate in many instances, especially in a remote community where there is a central organization, for example the research led by Jenefer Blackwell working with the Wurundjeri people [23], p25. However there is great variation in response, and sometimes one individual who is unwilling even to discuss a project, reduces the confidence in others and results in a negative response. In the area of western New South Wales where my own research has been centered there is no single discreet community but people live in towns with mixed populations. Consultation and negotiation since 1992 has proceeded through several organizations such as Wilcannia, Menindee, and Dareton Local Land Councils, Wilcannia Community Working Party, community health centers, Elders of the Mungo Joint Management Advisory Committee, and Maari Ma Health Aboriginal Corporation. In all letters that I have received between 1992 and 2012 that give support in principle, the organization does not give approval *per se* but recognizes and respects the right of individuals/families to continue participation. This is in keeping with the United Nations Declaration of the Rights of Indigenous Peoples [26] and the NH&MRC guidelines [27], p10 that state that

‘a community, organisation or person has the right to say “Yes” to be involved in research’, as well as having the right to say “no”.

How does this sit with the requirements of ‘gate-keeping’ committees, especially if they are comprised of people from a very broad region, not necessarily including representatives from a local area? Should a negative decision override that of participants and their families? Community organizational consent may not be appropriate in all communities especially where there are internal conflicts between powerful families over identity or resources. In my experience, as long as trusting relationships are in place, it is more acceptable to communities themselves, to be asked to ‘acknowledge’ that a research project has been negotiated and that the researchers are proceeding respectfully with willing individuals/families.

• Discomfort with the need for publication and the sharing of data, even though it is anonymized, is a frequent cause for delay. Clearly, the focus of population biologists and forensic scientists cross paths here, but under present guidelines it is difficult to collaborate in Australia. It is currently the reason why a recent publication using samples for which I remain custodian [8] has not made raw data files of a genome wide study available to other researchers. The general fear is that data (and sample) sharing might result in misuse and violate approval that has been given for specific studies. For all parties this sets a limit on the value of results and is contrary to the academic principles that see the sharing of research outcomes as fundamental to the growth of scientific knowledge. This point is highly relevant to the common interests shared by researchers in the current era of genome sequencing. Can projects really be presented as focused on a few specific questions? Even if there is trust between researchers and researched and the scale and purpose of a project is fully understood, results of genome research offer the opportunity to interrogate the genome to answer different questions whether they be medical/health, forensic or ancestry-related questions. Restricted access databases that require a user to declare acceptance of the ethical principles underlying the consent process offer one way forward, but no agreement has yet been reached to put this in place.

• Ancestry-focused genetic research is important to understand evolution of wellness/illness as well as history. However, for some, it is seen as a threat to traditional belief systems. It follows from impositions of other religions during colonization. Many Aboriginal and Torres Strait Islander people retain a strong traditional belief system combined with Christianity, and in the area in which I work, accept the genetic results into their heritage acquired through archaeological and social science [6]. Aboriginal people from the strong language groups of the area are involved in archaeology, management of the Willandra World Heritage Area, health, housing, and education, and express interest in research including the genetic work. There are for a few, fears that genetic research might produce results that erode identity, especially where colonization has imposed separation of families, mixed partnerships, and forced relocation. This is expressed as a fear that Native Title claims might be threatened by genetic research. There is no such requirement as part of the criteria for Native Title claimants [28] and it would not be appropriate as genetic connections of the past do not necessarily equate with the social and cultural connections that resulted in the language groups in specific country at the time of European occupation.

In summary, my view is that geneticists should not be daunted by the complex process one is required to go through to include Aboriginal and Torres Strait Islanders in genetic studies. There is currently no other choice but to build effective partnerships and broaden the dialogue with a goal of achieving beneficial outcomes for genetic researchers and researched. To do this, scientists have to be prepared to invest more than their expertise in genetics to work with Aboriginal and Torres Strait Islander peoples. They need to build appropriate teams of personnel, nurture relationships, and develop better ways of communicating their intentions and the outcomes of research so that participants can see the benefits that might flow without infringing human rights. They also need to be prepared for delays and possible refusal but only with perseverance and honesty will collaborative relationships be forged. The message has to be clear that, unlike practices of the past where indigenous peoples were patronisingly singled out as ‘interestingly different’, the focus is to include and not to exclude them from what should be research that recognizes their survival, resilience, and the role of genetics in shaping wellness.

## Competing interests

The author declares no competing interests.

## Authors’ information

The author is currently a Visiting Research Fellow in the School of Biotechnology and Biomolecular Sciences at the University of New South Wales, Sydney Australia. Prior to 2005, research was conducted at the University of Sydney, Australia. Formal studies included anthropology, archaeology, and genetics. Academic and research experience included Australian Aboriginal studies, Aboriginal health and education, medical research, nursing. Genetic research with Aboriginal Australians from the Darling River region of western New South Wales began in 1992 and included extensive ongoing consultation with the communities in the region. Ethical approval to continue work is currently being considered.
